# The Vanishing Stenosis: ST Elevation Myocardial Infarction and Rhythm Disturbance due to Coronary Artery Spasm—Case Report and Review of the Literature

**DOI:** 10.1155/2010/132902

**Published:** 2010-03-25

**Authors:** J. Gosai, C. J. Malkin, E. D. Grech

**Affiliations:** Department of Cardiology, Sheffield Teaching Hospitals NHS Foundation Trust, Northern General Hospital, Sheffield S5 7AU, UK

## Abstract

A 62-year-old lady was admitted with clinical and electrocardiograph features of acute myocardial infarction. Urgent coronary arteriography was performed, demonstrating a single discrete stenosis of one coronary artery. Following intracoronary injection of GTN, this stenosis completely resolved, as the symptoms did. The causes of acute myocardial infarction with normal coronary arteries are reviewed.

## 1. Case Presentation

**Figure 1:**
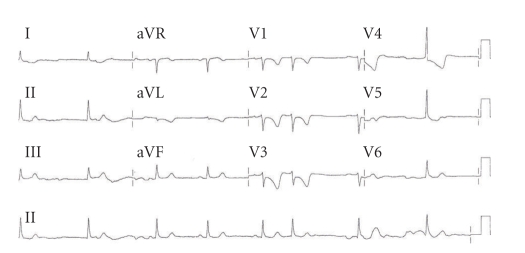
ECG demonstrating Inferior ST elevation, T wave inversions.

A 62-year-old lady with a history of treated hypothyroidism and obesity presented with a 12 hour history of severe central chest pain radiating to her left arm and not responding to sublingual nitrates. On arrival, she appeared in severe pain, and electrocardiogram (ECG) showed intermittent heart block and ST elevation in leads II, III and aVF with ST depression and deep *T* wave inversions in leads V2—V5 (Figure 1). A diagnosis of infero-posterior ST elevation myocardial infarction was made and she was transferred to the cardiac catheterisation suite for primary angioplasty.

The patient had no known risk factors for coronary artery disease except for obesity (BMI 44). She had previously had a diagnosis of ischaemic heart disease and angina and previous ECGs had shown T wave inversion in the inferolateral leads. There had been extensive correspondence about her problematic exertional chest pain and non-compliance with therapy.

**Figure 2:**
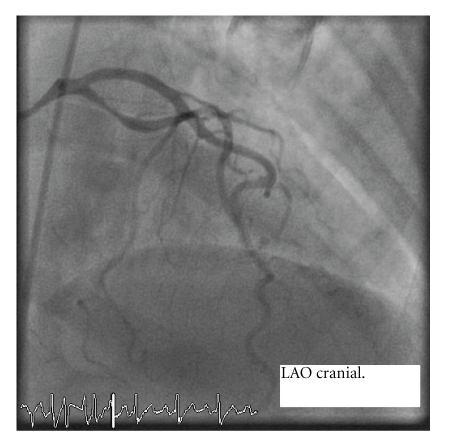


Coronary angiography showed an 80% discrete stenosis of the proximal circumflex artery (Figure 2).

**Figure 3:**
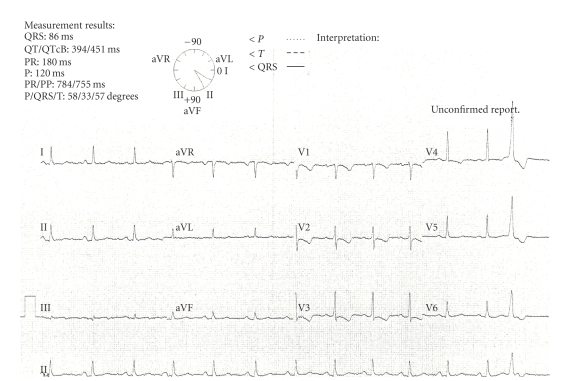
ECG immediately post angiography and IC GTN.

**Figure 4:**
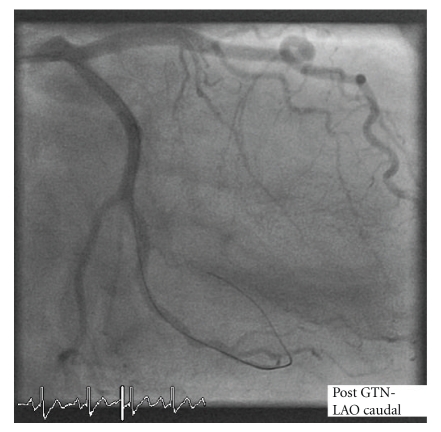


Initially the circumflex lesion was crossed with a hydrophilic floppy guide wire, and 200 micrograms intracoronary glyceryl trinitrate (GTN) was administered. Following this, there was an immediate and complete resolution of the apparent luminal stenosis, with marked increase in the calibre of the coronary artery throughout. The ECG and symptoms immediately improved (Figures 3 and 4).

The patient was treated overnight with intravenous GTN infusion and converted to a long acting oral nitrate preparation. She had no further symptoms and troponin I was measured at 0.07 ng/mL at 12 hours post pain (normal range <0.05). The patient was discharged. At 6 months she was reported to have few ongoing symptoms and improved exercise tolerance.

## 2. Discussion

Coronary spasm as a mimic of acute coronary syndrome including acute myocardial infarction (AMI) is a well documented phenomenon, associated with chest pain, hospital admissions and treatment as for atherosclerotic heart disease including angiography. It can also cause life threatening arrhythmia and other complications related to ischaemic myocardium. 

Coronary spasm may occur in normal coronary arteries, but is more common in vessels with non-obstructive atheroma which do not cause significant flow limitation at angiography but reduce the reserve capacity for flow through the vessel. The prevalence is higher in Japanese and Korean populations than in the Western population, probably due to a combination of genetic and environmental factors. Polymorphisms of the endothelial nitric oxide synthase and angiotensin II type 1 receptor have been put forward as pro-vasospastic with reduced endothelial nitric oxide activity and increased inflammatory markers. Several authors have also suggested that smoking is a strong independent risk factor for coronary artery spasm. This spasm may be dynamic, and can involve both large and small coronary arteries, sometimes multiple. This may explain acute myocardial infarction without a definite 100% stenosis seen.

The recently reported CASPAR study by Ong et al. took a large series of patients presenting with chest pain progressing to coronary angiography. 28% (138 of 488) had no demonstrable culprit lesion, and of these 86% underwent acetylcholine provocation testing. 49% (*n* = 42) demonstrated evidence of significant coronary spasm defined as a reduction in vessel calibre of at least 75% [1].

Animal models have demonstrated upregulation of coronary artery Rho-Kinase (ROCK) activity, resulting in increased myosin light chain phosphorylation (via myosin light chain phosphatase inhibition). An abnormal vasoconstrictor response to acetylcholine is seen. This forms the basis of the ergonovine provocation test used to assess propensity to vasospasm in those with chest pain and non flow limiting disease at angiography. 

Martínez-Sellés et al. demonstrated ergonovine induced spasm in 20% of 346 patients who had presented with chest pain and normal coronary arteries, and that inducible vasospasm was a positive predictor for future presentation with vasospasm. However the same authors also found that the baseline ECG and any ECG changes seen did not correlate with the presence of vasospasm. Hattori et al. also describe cases of an ergonovine provocation test producing vasospasm, and subsequent delivery of GTN failing to reverse or possibly even exacerbating this [2–6].

Intense vasospasm is seen in AMI following cocaine use (and certain amphetamine like compounds such as Catha edulis—khat), capecitabine administration, ethanol intoxication and following abrupt withdrawal of calcium channel blocking drugs. The alpha-adrenoceptor agonist effects of cocaine were thought to be the primary mechanism for cocaine induced coronary spasm, although it seems likely that this occurs in individuals predisposed to coronary spasm. There are case reports of pseudoephedrine induced acute myocardial infarction with normal coronary arteries in those using the drug for viral upper respiratory tract infections [5, 7–9] (see Table 1).

The anti-migraine triptan drugs are contra-indicated in those with known atherosclerotic coronary artery disease, and are though to induce vasoconstriction. However a recent study to quantify this showed only a modest vasoconstrictive effect of up to 6.8% of coronary artery diameter with two different triptan drugs in those with stable angina [10].

Unsurprisingly, associations have been made between Raynaud's disease, or limited systemic sclerosis (scleroderma) and coronary artery spasm, as well as one study finding an increased prevalence of migraine in those with coronary vasospasm (but not an increased prevalence of Raynaud's). As such, in one case report, iloprost was used as therapy and appeared to reverse the coronary spasm. In another, an emotional upset was thought to have triggered the event in a patient with CREST and acquired protein S deficiency. One small study of patients with documented coronary artery spasm demonstrated reproducible myocardial perfusion defects when the patients were exposed to the cold. Control subjects with atherosclerosis did not. The defects were reversed on rewarming [11–13].

Other associations with coronary artery spasm are hyperthyroidism (classically described in young Asian women and resolving on establishing euthyroidism). There are also a two case reports of hypothyroidism associated with Raynaud's phenomenon and coronary artery spasm [14–18].

In the acute setting, nitrates are the preferred treatment for coronary spasm, although efficacy is variable. Rarely other interventions are required in the context of spasm causing myocardial infarction. 

Maintenance therapy is based on nitrates and calcium channel blockers. Ninomiya et al. demonstrated in a small study of patients on either long term long acting nitrates or calcium channel blocker with anginal symptoms and normal or near normal coronary arteries and segments displaying a vasoconstrictive response to acetylcholine that the degree of flow restriction when challenged with acetylcholine was greater in the nitrate group than the calcium channel blocker group. The conclusions were that treatment with calcium channel blockers may be more effective than nitrates in the long term. Beta blockers should be avoided, as there is a risk of precipitating spasm with these [19].

**Learning Points**

(i) Coronary artery spasm well is a documented cause of chest pain and presentation as acute coronary syndrome. (ii) Coronary arteriography may demonstrate normal arteries or disease at a level inconsistent with symptoms. The stenosis may be dynamic, involving large or small and often multiple coronary arteries. (iii) It can cause acute myocardial infarction, including sudden death. (iv) Treatment acutely is with nitrate. (v) Treatment acutely is with nitrate. (vi) It is most commonly seen in Asian populations, young women and smokers. (vii) Other associations include drugs, thyroid dysfunction, and scleroderma. The underlying pathophysiology is unclear but thought to be EDRF related. (ix) Maintenance treatment is with calcium channelblocking drugs or nitrates. Beta blockers are best avoided.

## Figures and Tables

**Table 1:** Associations of coronary artery vasospasm.

Smoking	
Thyroid dysfunction	

Raynaud's phenomenon	

Scleroderma	

Cold exposure	

Migraine (conflicting evidence)	

Drugs	Cocaine

	Ethanol

	Amphetamines

	Calcium channel blockers
	(withdrawal)

	Capecitabine
